# Inactivation of beta1 integrin induces proteasomal degradation of Myc oncoproteins

**DOI:** 10.18632/oncotarget.27131

**Published:** 2019-08-13

**Authors:** Manabu Sasada, Takuya Iyoda, Tatsufumi Asayama, Yusuke Suenaga, Shunsuke Sakai, Naoya Kase, Hiroaki Kodama, Sana Yokoi, Yoichiro Isohama, Fumio Fukai

**Affiliations:** ^1^ Department of Molecular Pathophysiology, Faculty of Pharmaceutical Sciences, Tokyo University of Science, Chiba, Japan; ^2^ Translational Research Center, Research Institute of Science and Technology, Tokyo University of Science, Chiba, Japan; ^3^ Laboratory of Applied Pharmacology, Faculty of Pharmaceutical Sciences, Tokyo University of Science, Chiba, Japan; ^4^ Cancer Genome Center, Chiba Cancer Center Research Institute, Chiba City, Chiba, Japan; ^5^ Department of Pharmacy, Faculty of Pharmaceutical Sciences, Sanyo-Onoda City University, Sanyo-Onoda City, Yamaguchi, Japan; ^6^ Faculty of Science and Engineering, Saga University, Saga, Japan

**Keywords:** integrin, Myc, proteasomal degradation, fibronectin, neuroblastoma

## Abstract

The *MYC* family oncogenes (*MYC*, *MYCN*, and *MYCL*) contribute to the genesis of many human cancers. Among them, amplification of the *MYCN* gene and over-expression of N-Myc protein are the most reliable risk factors in neuroblastoma patients. On the other hand, we previously found that a peptide derived from fibronectin, termed FNIII14, is capable of inducing functional inactivation in β1-integrins. Here, we demonstrate that inactivation of β1-integrin by FNIII14 induced proteasomal degradation in N-Myc of neuroblastoma cells with *MYCN* amplification. This N-Myc degradation by FNIII14 reduced the malignant properties, including the anchorage-independent proliferation and invasive migration, of neuroblastoma cells. An *in vivo* experiment using a mouse xenograft model showed that the administration of FNIII14 can inhibit tumor growth, and concomitantly a remarkable decrease in N-Myc levels in tumor tissues. Of note, the activation of proteasomal degradation based on β1-integrin inactivation is applicable to another Myc family oncoprotein, c-myc, which also reverses cancer-associated properties in pancreatic cancer cells. Collectively, β1-integrin inactivation could be a new chemotherapeutic strategy for cancers with highly expressed Myc. FNIII14, which is a unique pharmacological agent able to induce β1-integrin inactivation, may be a promising drug targeting Myc oncoproteins for cancer chemotherapy.

## INTRODUCTION

The Myc family of oncogenes encode three kinds of transcription factor proteins (c-, N-, and L-myc) that are involved in physiological cellular processes, including cell proliferation and several metabolic pathways [1, 2]; their expression is strictly controlled in normal cells [3]. However, Myc proteins, once they are excessively expressed, cause a variety of cancer-associated aggressive properties, such as dysregulated survival and proliferation and drug-resistance in cancer cells [1, 2, 4, 5]. Among the Myc family of oncoproteins, over-expression of N-Myc is restricted in some cancers, including neuroblastoma, a common pediatric cancer. Neuroblastoma patients with the *MYCN* gene amplification are classified into the high-risk group, and their survival rates remain in the range of 30%–40%, which represents the highest number of cancer-related deaths among pediatric solid cancers [6, 7]. On the other hand, over-expression of c-myc is found in a variety of cancers, such as small cell lung cancer and pancreatic cancer, and plays a crucial role in their pathogenesis [8–10]. Thus, the Myc family of oncoproteins are considered to be important target molecules for cancer chemotherapy [2, 4, 7]. However, attempts to develop a drug targeting the Myc family of oncoproteins have failed because they are non-enzymatic transcription factors lacking a specific active site for small molecules, thus making it difficult to inhibit their activity [11, 12].

Cell adhesion to the extracellular matrix (ECM), mainly mediated by β1-integrins, is a critical event for cell regulation, including survival, proliferation, differentiation, and gene expression [13–15]. We previously found that fibronectin harbors a cryptic functional site within the molecular structure [16, 17]. A peptide derived from fibronectin, termed FNIII14, is capable of inducing conformational change in β1-integrins necessary for their functional inactivation. FNIII14 greatly influences the anchorage-dependent cellular functions through negative regulation of β1-integrin-mediated cell signaling [18–20].

Here, we demonstrate that inactivation of β1-integrin by FNIII14 induced proteasomal degradation in N-Myc of human neuroblastoma cells with the *MYCN* gene amplification. This N-Myc protein degradation attenuated the cancer-associated malignant properties of neuroblastoma cells. An *in vivo* experiment using a mouse xenograft model showed the therapeutic potential of FNIII14 for aggressive neuroblastoma. Importantly, integrin inactivation-based proteasomal degradation was shown to be applicable to another MYC family oncoprotein, c-myc, which is over-expressed in a variety of malignant tumors, such as small cell lung cancer and pancreatic cancer. The present study provides insights into the potential pharmacological application of FNIII14 as an antitumor agent against cancers with highly expressed Myc oncoproteins.

## RESULTS

### FNIII14 induces a remarkable decrease in N-Myc protein levels in neuroblastoma cells based on proteasomal degradation

**Figure 1:**
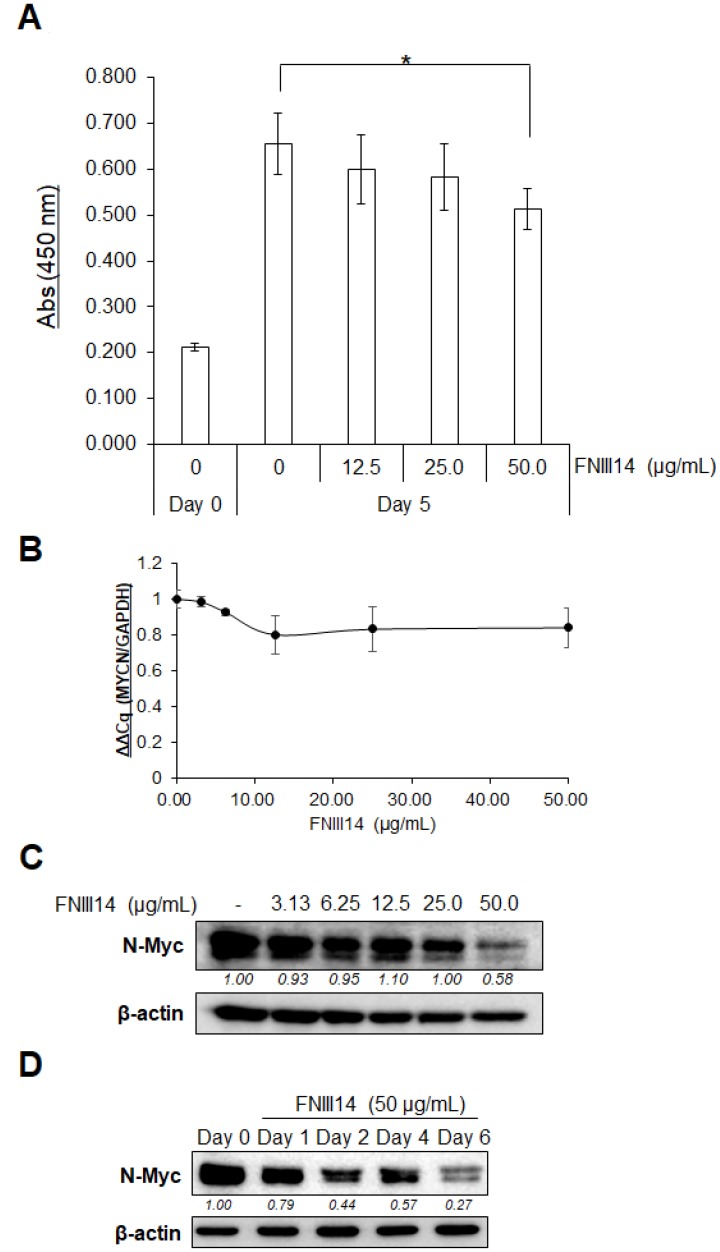
FNIII14 induces a reduction of N-Myc protein levels. (**A**) Effect of FNIII14 for proliferation of IMR-32 cells. IMR-32 cells adhered on the fibronectin substrate were stimulated with or without FNIII14 (50 μg/mL) for 5 days and then subjected to the WST assay, as described in ‘Materials and Methods’. Each point represents the mean ± S.D. of triplicate determinations, ^*^ *P* < 0.05. (**B** and **C**) Effect of FNIII14 on the expression of MYCN gene and N-Myc protein. IMR-32 cells were stimulated with the indicated concentrations of FNIII14 for 6 days and then subjected to real-time PCR (B) or Western blot analysis (C). (**D**) Time-dependent decrease in N-Myc protein levels by FNIII14. IMR-32 cells were stimulated with FNIII14 (50 μg/mL) for the indicated days and then subjected to Western blot analysis to detect N-Myc protein. In (C) and (D), the intensity of the immunoblot was quantified densitometrically and represented as relative intensity.

IMR-32 cells were used in this study as a human neuroblastoma cell line with *MYCN* gene amplification. Similar to our previous results using various cell types [18, 20], FNIII14 also weakened the adhesion of IMR-32 cells to the fibronectin substrate (Supplementary Figure 1A) through inactivation of β1-integrins (Supplementary Figure 1B). We then examined the effect of the FNIII14-induced β1-integrin inactivation on IMR-32 cell growth on the fibronectin substrate. When IMR-32 cells were cultured for 5 days in the presence or absence of FNIII14, IMR-32 cell proliferation was suppressed in a dose-dependent manner (Figure 1A). Since N-Myc protein serves as a transcription factor that activates the expression of pro-proliferative genes [21], we examined the effect of FNIII14 treatment on the expression of the *MYCN* gene and N-Myc protein. As shown in Figure 1B, no significant change was detected in the expression of *MYCN* gene by FNIII14 when evaluated by real-time PCR. In contrast, western blot analysis showed a remarkable decrease in N-Myc protein level after FNIII14 treatment (Figure 1C). This decrease in N-Myc protein levels by FNIII14 was time-dependent and a marked decrease was observed after 6 days of incubation (Figure 1D). The FNIII14-induced decrease in N-Myc protein was reproducible in other human *MYCN*-amplified neuroblastoma cell lines, such as NB-1 cells and KELLY cells. (Supplementary Figure 2).

**Figure 2:**
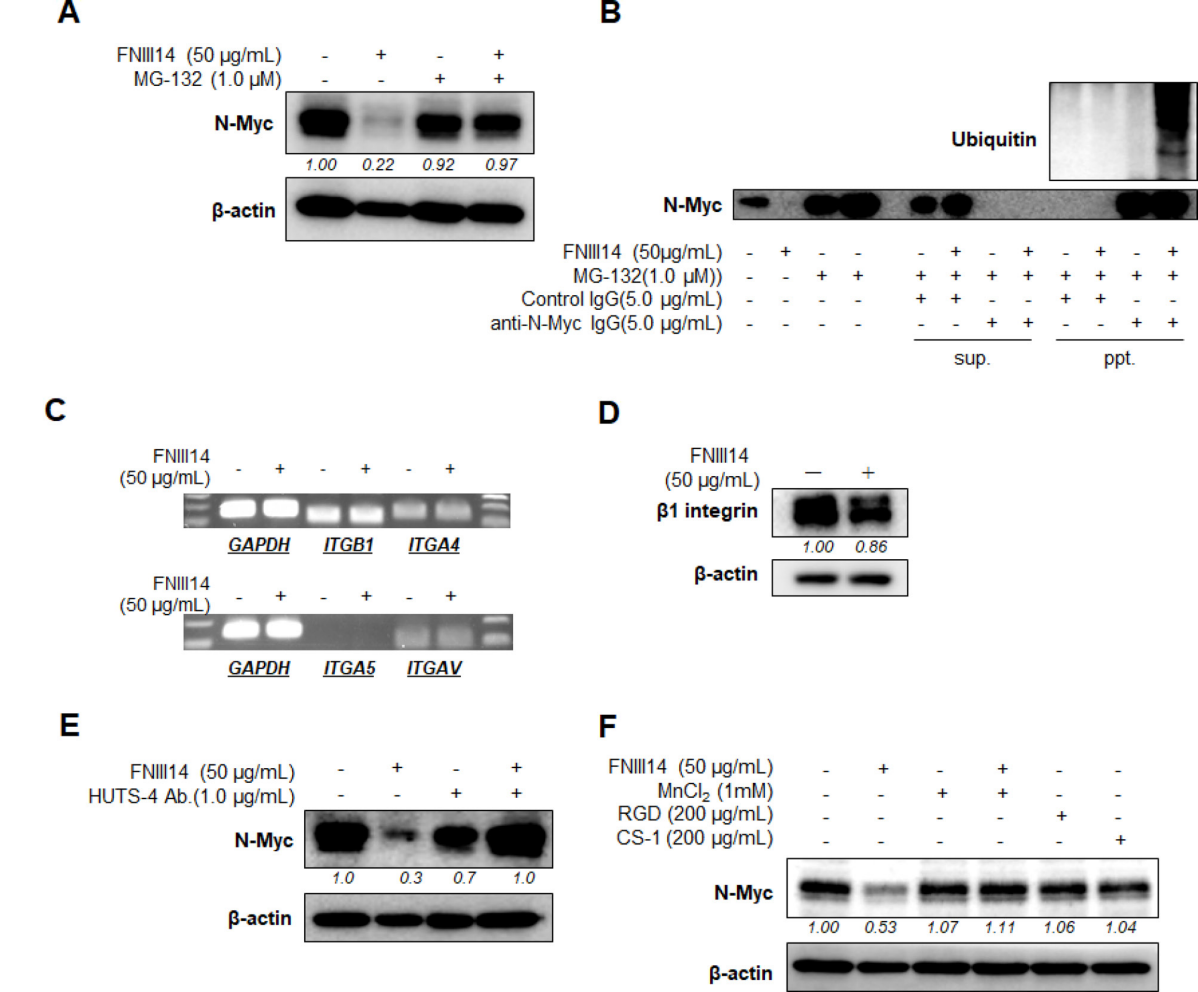
FNIII14 induces proteasomal degradation of N-Myc protein through inactivation of β1-integrins. (**A** and **B**) Involvement of proteasomal degradation in FNIII14-induced decrease in N-Myc protein. IMR-32 cells were cultured in the presence or absence of FNIII14 (50 μg/mL). MG-132, a proteasome inhibitor, was added to this culture on day 5, and cell lysates on day 6 were subjected to Western blot analysis using N-Myc Ab (A). Immunoprecipitation study using anti-N-Myc Ab was performed in (B), as described in “Material and Methods”. Cell lysates were also subjected to immunoblot analysis to detect ubiquitination of N-Myc protein. (**C**) Effect of FNIII14 on gene expression of integrin subunits. IMR-32 cells stimulated with or without FNIII14 (50 μg/mL) for 6 days were subjected to RT-PCR to detect integrin gene of β1 (ITGB1), α4 (ITGA4), α5 (ITGA5) and αv (ITGAV). (**D**) Effect of FNIII14 on integrin β1 protein expression. IMR-32 cells treated as in (C) were subjected to Western blot analysis to detect N-Myc protein. (**E** and **F**) Involvement of β1-integrin inactivation in FNIII14-induced N-Myc protein degradation. IMR-32 cells were stimulated with or without FNIII14 (50 μg/mL) in the presence or absence of factors that affect the β1-mediated cell adhesion: HUTS-4 = β1-integrin-activating mAb, MnCl2 = integrin activator, RGD and CS-1 = antagonistic peptides for integrin αvβ1 and α4β1, respectively. After 6 days of culture, cells were subjected to Western blot analysis using anti-N-Myc Ab. In (A) and (D–F), the intensity of the immunoblots was quantified densitometrically and represented as relative intensity.

To examine the involvement of targeted degradation in the FNIII14-induced decrease of N-Myc, we tested the effect of MG-132, a proteasome inhibitor, on the decrease of N-Myc. As shown in Figure 2A, the decrease in N-Myc protein level induced by FNIII14 was completely canceled by the addition of MG-132. Moreover, an immunoprecipitation study using anti-N-Myc antibody (Ab) further showed that FNIII14 treatment markedly elicited ubiquitination of N-Myc in IMR-32 cells (Figure 2B). Thus, FNIII14 was shown to induce N-Myc protein degradation through activation of the ubiquitin-proteasome system.

### Molecular basis of the proteasomal degradation of N-Myc protein induced by FNIII14

We investigated the involvement of integrins on the FNIII14-induced N-Myc degradation. First, we examined whether the expression of integrin with high affinity to fibronectin was altered in the course of FNIII14 treatment. As shown in Figure 2C, clear β1 gene expression was observed and that of its counterparts α4 and αv but α5 was not detected. RT-PCR showed that gene expression of integrin subunits, α4, αv, and β1, were barely changed by FNIII14 treatment (Figure 2C). Because FNIII14 was found to be a factor that inactivates β1-integrins [18], β1 subunit was also analyzed at the protein level. As shown in Figure 2D, the expression levels of the β1 subunit detected by western blotting was not altered by FNIII14.

We next investigated whether inactivation of β1-integrins was responsible for the proteasomal degradation of N-Myc induced by FNIII14. The effects of two different factors, which are able to activate β1-integrins, were examined. Divalent metal ions including Mn^2+^ [22] are known to widely activate integrins [22], and HUTS-4, a monoclonal antibody (mAb) that recognizes the active conformation-specific epitope, can specifically activate β1-integrins [22, 23]. As shown in Figure 2E and 2F, the FNIII14-induced decrease in N-Myc protein level was reversed by the addition of either MnCl_2_ or HUTS-4, suggesting that β1-integrin inactivation was responsible for the FNIII14-induced proteasomal degradation of N-Myc. On the other hand, treatment with the peptides CS-1 and GRGDSP, which are specific antagonists for the fibronectin receptors expressed on IMR-32 cells, such as integrin α4β1 and αvβ1, respectively, barely influenced N-Myc protein levels (Figure 2F). These results thus indicated that the conformational and consequent functional inactivation of β1-integrins, but not the prevention of β1-integrin ligation by integrin antagonists, is essentially required to induce the proteasomal degradation of N-Myc protein.

**Figure 3:**
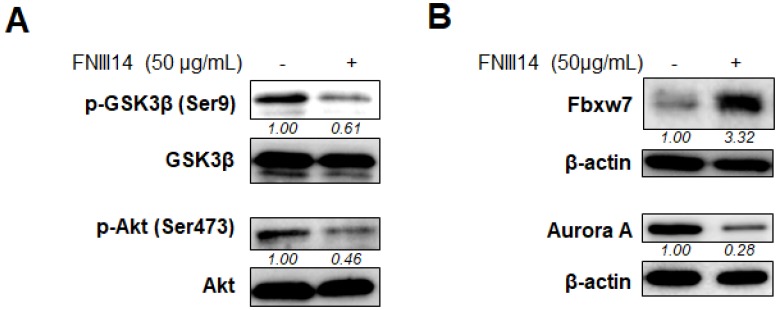
Molecular basis of proteasomal degradation of N-Myc protein induced by FNIII14. IMR-32 cells adhered on the fibronectin substrate were cultured with or without FNIII14 (50 μg/mL) for 6 days. Cell lysates were subjected to Western blot analysis. (**A**) Effects of FNIII14 for phosphorylation levels of GSK3β at Ser9 and Akt at Ser473. (**B**) Effects of FNIII14 for intracellular level of Fbxw7 protein and Aurora A protein. The intensity of the immunoblot was quantified densitometrically and represented as relative intensity.

The signaling pathway for triggering the proteasomal degradation of N-Myc protein was investigated. Analysis of the signaling pathway downstream of β1-integrin showed that β1-integrin inactivation by FNIII14 was accompanied by the dephosphorylation of Akt at Ser473 and GSK3β at Ser9 (Figure 3A), both of which are known to serve as a driving signal to enhance the proteasomal degradation of N-Myc by facilitating the ubiquitination of N-Myc [24]. We next examined the protein expression of Fbxw7, an E3-ubiquitin ligase for N-Myc protein [25, 26], by inducing targeted degradation *via* the ubiquitin proteasome system. As shown in Figure 3B, IMR-32 cells treated with FNIII14 upregulated the expression level of Fbxw7. Additionally, FNIII14 treatment also induced downregulation in the expression of Aurora A (Figure 3B), which inhibits proteasomal degradation by binding to N-Myc protein [25]. These results showed that β1-integrin inactivation by FNIII14 accelerated the proteasomal degradation of N-Myc by promoting not only ubiquitination of N-Myc, but also its access to the proteasome complex.

### FNIII14-induced N-Myc protein degradation causes attenuation of cancer-associated malignant properties of neuroblastoma cells

**Figure 4:**
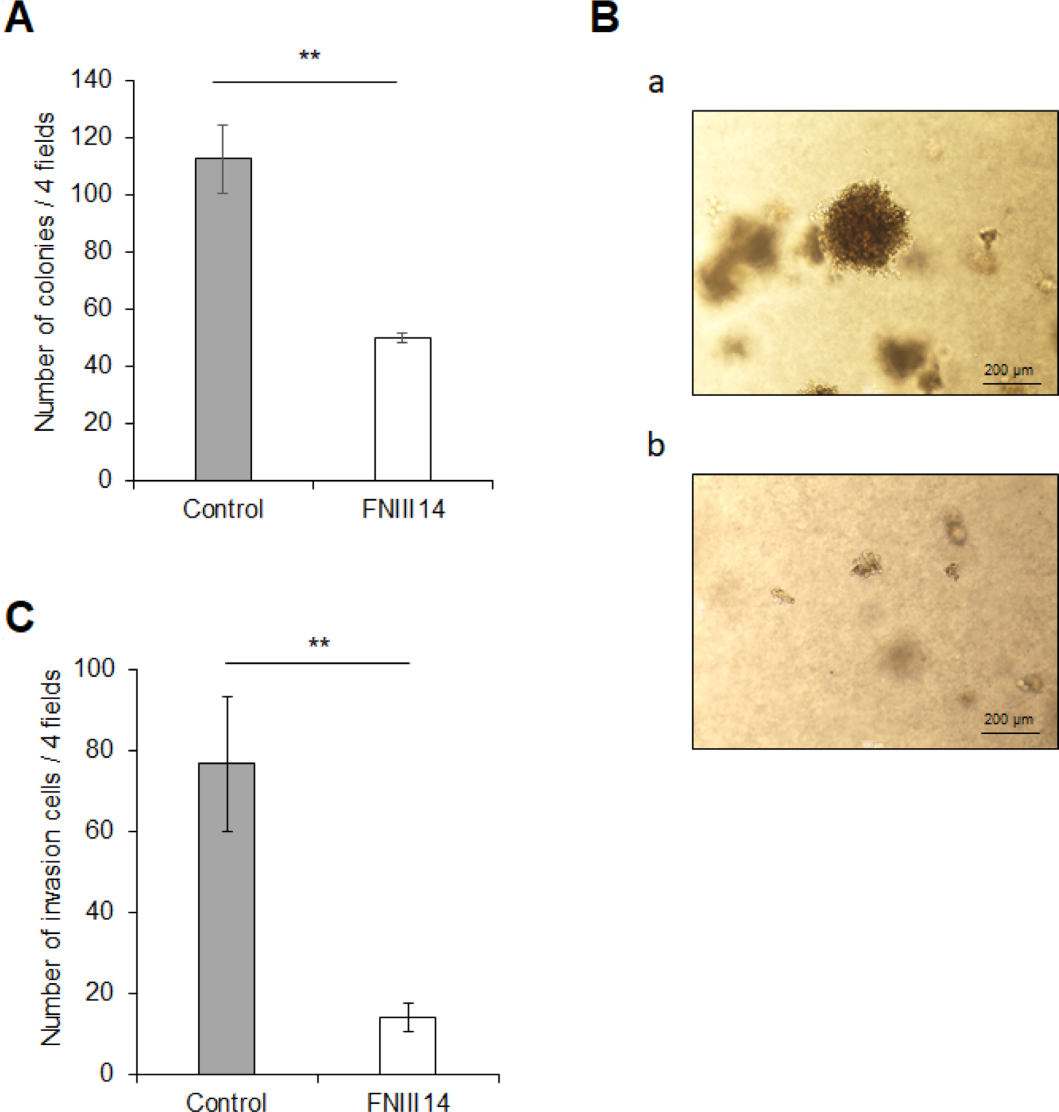
FNIII14 has the ability to attenuate cancer-related malignant properties. (**A** and **B**) Effect of FNIII14 on anchorage-independent cell growth. IMR-32 cells treated with (gray bar) or without FNIII14 (open bar) for 6 days were subjected to the colony formation assay as described in ‘Materials and Methods’. (A): Number of colonies formed after 3 weeks. (B): Representative images of colonies in “Control” (without FNIII14) (a) and “FNIII14” (with FNIII14) (b). (**C**) Effect of FNIII14 on the invasion ability of IMR-32. IMR-32 cells treated with (gray bar) or without FNIII14 (open bar) for 6 days were subjected to the *in vitro* invasion assay as described in ‘Materials and Methods’. Each point represents the mean ± S.D. of triplicate determinations, ^**^ *P* < 0.01.

Excessive expression of N-Myc contributes to the cancer-associated malignant properties of high-risk neuroblastoma cells [4, 27, 28]. Therefore, FNIII14-induced N-Myc protein degradation is expected to cause attenuation of the malignant properties in neuroblastoma cells. As shown in Figure 4A, untreated cells grew in soft agarose and more than 100 colonies were formed, but the cells treated with FNIII14 showed attenuated ability to grow in soft agarose, and the number of colonies represented less than half the untreated cells. An *in vitro* invasion assay also showed that FNIII14 treatment suppressed the ability of invasion through the basement membrane-like matrix EHS-gel (Figure 4B).

**Figure 5:**
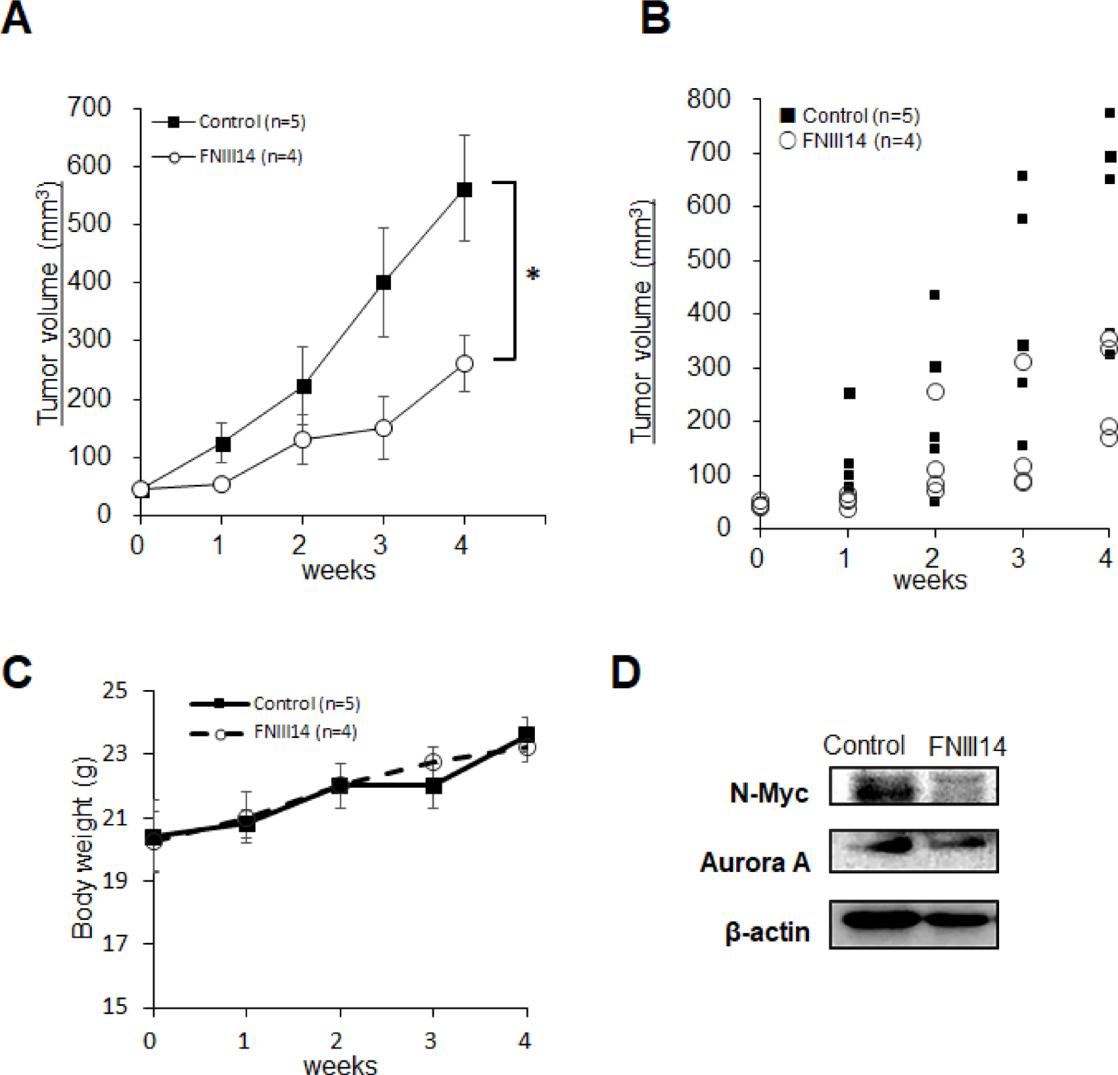
Administration of FNIII14 suppresses tumor growth in neuroblastoma xenograft model. IMR-32 cells (2.0 × 10^6^ cells/head) suspended in MEM containing EHS-gel were subcutaneously injected into the left flank of Balb/c nude mice. Mice bearing established tumors were divided into two groups: the Control group was administered vehicle and the FNIII14 group was administered peptide FNIII14, as described in the Materials and Methods. Chemotherapy was initially carried out for 1 week. Tumor volume (mm^3^) was determined by measuring with calipers and calculated according to hemi-ellipsoid model: Volume = 2/3 × π × (major axis/2) × (minor axis/2)^2^. Three weeks after chemotherapy, tumors removed from mice were subjected to western blot analysis to detect N-Myc and Aurora A as described in the ‘Materials and Methods’. (**A**) Tumor growth curves for IMR-32 xenografts (Control group, *n* = 5; FNIII14 group, *n* = 4). Tumor volumes are shown as means ± S.E. Data was analyzed by Mann-Whitney *U*-test. (**B**) Representative dot plots showing the tumor volume for each week. (**C**) Body weight of each group. (**D**) Expression of N-Myc and Aurora A proteins in tumor tissues of the Control and FNIII14 groups.

Based on these *in vitro* results, the therapeutic potential of FNIII14 was tested using a mouse xenograft model for human high-risk neuroblastoma. After transplanting IMR-32 cells, mice bearing tumors were divided into two groups: the control-group was administered with vehicle and the FNIII14-group with the peptide FNIII14. Mice were injected daily with vehicle or FNIII14 for one week and then monitored for tumor growth. After monitoring for 5 weeks, tumors were removed, and their lysates were subjected to western blot analyses of N-Myc protein levels. As shown in Figure 5A and 5B, the administration of FNIII14 significantly delayed tumor growth even though chemotherapy lasted only one week. This tumor growth inhibition was in parallel with a marked decrease in N-Myc protein levels in tumor tissues (Figure 5D). No significant weight loss or tissue damage was observed in mice after the administration of FNIII14 (Figure 5C). These results indicated that FNIII14 has a therapeutic potential for neuroblastomas with *MYCN* gene amplification.

### Proteasomal degradation based on integrin-inactivation is applicable for degradation of c-myc protein.

**Figure 6:**
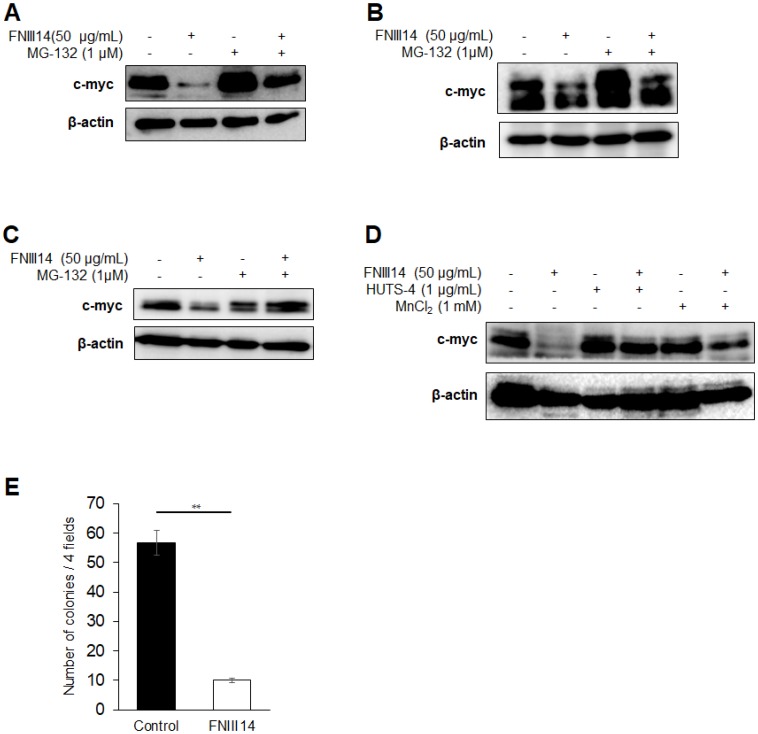
FNIII14 is able to induce degradation of c-myc as well as N-Myc. (**A**–**C**) Induction of proteasome-dependent degradation of c-myc protein by FNIII14. Various cancer-derived cell lines (Pancreatic cancer MIA-PaCa 2 cells in (A), small cell lung cancer NCI-H82 cells in (B) and chronic myelogenous leukemia K562 cells in (C)) were cultured with each medium in the presence or absence of FNIII14 in the same manner as in the case of neuroblastoma cells IMR-32. MG-132 was similarly added one day before the end of FNIII14 treatment and treated for 24 hours. (**D**) Involvement of β1-integrin inactivation in FNIII14-induced c-myc protein degradation. MIA-PaCa 2 cells were stimulated with FNIII14 in the presence or absence of factors capable of β1-integrin activation, HUTS-4 and MnCl2. (**E**) Effect of FNIII14 on the anchorage-independent growth of MIA-PaCa 2 cells. MIA-PaCa 2 cells were subjected to the colony formation assay as described in ‘Materials and Methods’.

The amino acid sequences of the Myc family of proteins, especially the Myc box domain involved in proteasomal degradation [11], are highly conserved between each protein. This prompted us to presume that β1-integrin inactivation by treatment with FNIII14 induces proteasomal degradation in another Myc family protein, c-myc, which is highly expressed in a wide variety of cancers. The pancreatic cancer cell line MIA-PaCa 2 and small cell lung cancer cell line NCI-H82 were used as cancer cells with high expression of c-myc protein. FNIII14 treatment of MIA-PaCa 2 cells induced a significant decrease in c-myc protein level and was canceled by the addition of MG-132 (Figure 6A). Similarly, FNIII14 treatment decreased the c-myc protein level in NCI-H82 cells and K562 cells; this change was abrogated by MG-132 (Figure 6B and 6C). Moreover, the addition of factors capable of activating β1-integrin, namely, HUTS-4 Ab and MnCl_2_, abrogated the effect of FNIII14 (Figure 6D). FNIII14 treatment also attenuated the ability of MIA-PaCa 2 cells to grow in soft agarose (Figure 6E). It was thus demonstrated that β1-integrin inactivation by FNIII14 was also responsible for proteasomal degradation of c-myc protein, which caused attenuation of the cancer-associated malignant properties.

Collectively, β1-integrin inactivation seems to be common signaling that induces proteasomal degradation of Myc family oncoproteins, and consequently causes a significant reduction in the cancer-associated malignant properties. FNIII14, which is a unique peptide factor capable of inactivating β1-integrins, may be useful for chemotherapeutic treatment of malignant tumors with high expression of Myc oncoproteins.

## DISCUSSION

The Myc family of oncoproteins plays crucial roles in the cancer-associated aggressive feature of malignant tumors. Therefore, these oncoproteins have been implicated as important targets for cancer chemotherapy. However, there are no effective agents that directly target Myc oncoproteins. First, this is because the Myc proteins are non-enzymatic transcription factors that lack a specific active site for small molecules [12]. Second, because Myc proteins are present inside the cells, cytoplasm, and nuclei, direct targeting of these proteins by middle or large molecular strategies is problematic. In the present study, we demonstrated the induction of proteasomal degradation in N-Myc protein by inactivation of β1-integrins. Since FNIII14 markedly decreased the intracellular levels of N-Myc in aggressive neuroblastoma cells and remarkably reduced the cancer-associated malignant properties, FNIII14-induced β1-integrin inactivation could be considered to be functional for the targeting of N-Myc oncoprotein. In fact, an *in vivo* experiment using a xenograft model showed the therapeutic potential of FNIII14 for chemotherapeutic treatment of neuroblastoma. Notably, β1-integrin inactivation-based activation of the ubiquitin-proteasome system could be adopted for another Myc family protein, c-myc, which is highly expressed in a variety of cancers. Since the effect of FNIII14 is mediated by its receptor proteins present on cell surfaces [29], this peptide factor would be able to easily initiate the degradation of Myc oncoproteins independently of their subcellular localization. FNIII14, which is a unique pharmacological agent able to induce β1-integrin inactivation, may be promising as a drug that targets the Myc oncoproteins for chemotherapeutic treatment of cancers that show high expression of Myc oncoproteins.

Mechanistic analysis revealed that two different molecular pathways are functional in the FNIII14-induced proteasomal degradation of N-Myc. One route was in the Akt/GSK3β signaling pathway downstream of β1-integrin. As has been established, β1-integrin-mediated adhesive interaction induces Akt phosphorylation at Ser473 [30], which in turn suppresses the phosphorylation of Thr58 of N-Myc by GSK3β, resulting in prevention of the proteasomal degradation of Myc proteins. By inhibiting this pathway, FNIII14 successively stimulates the proteasomal degradation of Myc proteins. Another route is in the quantitative regulation of Fbxw7 and Aurora A expression levels. Fbxw7 is an E3 ligase that catalyzes ubiquitination of Myc family proteins, which facilitates the access to the proteasome complex [26], while Aurora A stabilizes Myc oncoproteins by inhibiting this molecular interaction [24]. Stimulation with FNIII14 not only upregulates the expression of Fbxw7, but also downregulates the expression of Aurora A in neuroblastoma cells. Thus, FNIII14 stimulated these different pathways concomitantly, making the degradation of N-Myc protein highly efficient. However, in contrast to the integrin/Akt/GSK3β pathway, the signaling pathway leading to changes in the expression of Fbxw7 and Aurora A, following the FNIII14-induced inactivation of β1-integrins has yet to be solved. Furthermore, recent studies demonstrate that additional factors, such as Ras/ERK, prolyl isomerase PIN-1, phosphatase PP2A, and PP2A inhibitor CIP2A/KIKA154 play important roles in the degradation and stabilization of Myc [31]. In order to elucidate the molecular mechanism of Myc degradation based on FNIII14-induced integrin inactivation, we must analyze the involvement of these functional molecules. A precise understanding of the signaling pathway would provide the molecular basis for the development of a more efficient cancer treatment targeting Myc oncoproteins.

This study demonstrated the particular importance of the inactivation of β1-integrins by FNIII14 for targeting Myc proteins. Since integrin-mediated cell adhesion to the ECM is essential for fundamental cell functions, including survival, proliferation, differentiation, and gene expression, β1-integrin inactivation by FNIII14 may exhibit strong cytotoxic effects on normal cells. However, cells in tissues adhere to a variety of ECM components by using several membrane receptors, such as the β2-6 subfamily of integrins and transmembrane proteoglycans. It seems that β1-integrin inactivation by FNIII14 is unable to induce cell detachment, but does induce de-adhesion, which is defined as the process involving the transition of the cell from a strongly adherent state to a state of intermediate adherence [32]. Indeed, our previous studies suggest that FNIII14 exhibits no significant injurious effects but is rather beneficial on the host. Matsunaga *et al.* reported that FNIII14 treatment in combination with an anti-cancer drug enables eradication of acute myelogenous leukemia with no significant myelosuppression by abrogating cell adhesion-mediated drug resistance [20]. Therefore, it can be expected that FNIII14 is applicable as an anti-tumor agent for various malignant tumors. However, to completely remove cancer cells from the body, FNIII14 therapy may need to be used in combination with additional chemotherapy in the form of general anti-cancer drugs. Further investigation into this possibility is now in progress.

## MATERIALS AND METHODS

### Reagents

Plasma fibronectin was purified from horse serum as described previously [33]. Peptide FNIII14 (TEATITGLEPGTEYTIYVIAL) has been described previously [16]. Peptide GRGDSP and peptide CS-1, which were used as antagonists for integrin α5β1 and integrin α4β1 integrin, respectively, were purchased from Eurofin genetics (Tokyo, Japan). MnCl_2_ 4H_2_O was purchased from Tokyo Chemical Industry Co., Ltd. (Tokyo, Japan). We purchased anti-human N-Myc Ab (OP13) and anti-Ubiquitin Ab (04-263) from Merck Millipore (Darmstadt, Germany), and anti-Aurora A Ab (#3092S), anti-Akt mAb (#9272S), anti-phospho Akt(Tyr473) mAb (#4051S), anti-GSK3β Ab (#9315S), anti-phospho GSK3β(Ser9) Ab (#9323S), and anti-c-myc Ab (#9402S) from Cell Signaling Technology Japan, K.K. (Tokyo, Japan). Anti-Fbxw7 Ab (ab109617) was purchased from Abcam (Tokyo, Japan). Anti-β1 integrin mAbs recognizing active conformation AG89 and HUTS-4 (MAB2079Z) were purchased from MBL Co., Ltd. (Nagoya, Japan) and Millipore, respectively. Proteasome inhibitor, MG-132 was obtained from Merck Millipore.

### Cell culture

Human neuroblastoma cell line IMR-32, which was obtained from JCRB cell bank, was maintained in MEM (41500-018; Gibco, Grand Island, NY) supplemented with 10% FBS and penicillin-streptomycin solution. Human pancreatic cancer cell line MIA-PaCa 2 which was kindly provided by Dr. Masuho (Tokyo University of Science), was maintained in DMEM (Nissui Pharmaceutical Co., Ltd., Tokyo, Japan) plus 10% FBS, 2 mM glutamine, penicillin-streptomycin solution, and 2.2 g/L NaHCO_3_. Human chronic myelogenous leukemia cell line K562 was obtained from the Resource Center for Biomedical Research, Institute of Development, Aging and Cancer, Tohoku University, and cultured with RPMI1640 medium plus 10% FBS, 2 mM glutamine, penicillin-streptomycin solution, and 2.2 g/L NaHCO_3._ Human small cell lung cancer cell line NCI-H82 was kindly provided by Dr. Makinoshima (National Cancer Research Institute, Tokyo, Japan) and cultured with RPMI1640 medium (Nissui Pharmaceutical Co., Ltd.) supplemented with 10% FBS, 2 mM glutamine, penicillin-streptomycin solution, and 2.2 g/L NaHCO_3_. Human neuroblastoma cell line NB-1 was obtained from ATCC, cultured with RPMI1640 medium plus 10% FBS, 2 mM glutamine, penicillin-streptomycin solution, and 2.2 g/L NaHCO_3_. Human neuroblastoma cell line KELLY was deposited from Sigma-Aldrich Japan (Tokyo, Japan), was maintained in RPMI1640 medium supplemented with 10% FBS, 2 mM glutamine, penicillin-streptomycin solution, and 2.2 g/L NaHCO_3_. Each experiment was carried out using thawed cells without further authentication. These cell lines were also authenticated by routine monitoring of cell morphology and proliferation, kept in a humidified incubator at 37° C with 5% CO_2_, and cultured up to 15 passages.

### Cell survival and proliferation

IMR-32 cells (5.0 × 10^3^ cells/well) were seeded on 96-well plates coated with fibronectin (2.0 μg/mL). The number of viable cells was evaluated by the WST-8 assay, as described previously [34].

### Colony formation assay

Solution of a 1:1 mixture of 1.4% Bacto-agar (BD Bioscience, Franklin Lakes, NJ) and 2× growth medium was poured into 12-well plates. After solidification, IMR-32 and MIA-PaCa 2 (5.0 × 10^3^ cells/well) suspended in growth medium containing 0.7% Bacto-agar in the presence or absence of FNIII14 were overlaid on top of a base layer. After solidification of the top agar layer, growth medium in the presence or absence of FNIII14 was added. Media were changed every week. After 3 weeks, colonies were stained with crystal violet and the number of cells was counted for five randomly selected fields under the microscope at 40× magnification. Each group was counted in 3 wells.

### Western blotting

IMR-32 cells (1.0 × 10^5^ cells/well) and MIA-PaCa 2 cells (8.0 × 10^4^ cells/well) were allowed to adhere in 6-well plates coated with fibronectin. Each treatment is described in the figure legends. Subsequent steps were conducted using antibodies shown among the reagents, as described previously [35].

### Immunoprecipitation

IMR-32 cells were cultured with or without FNIII14 (50 μg/mL) for 6 days. At 24 h before lysis, Cells were treated with or without MG-132 (1.0 μM). Cells were dissolved with the RIPA buffer (25 mM Tris, 15 mM NaCl, 1.0 mM EDTA, 1.0% NP-40, 5.0% glycerol, pH 7.4) and prepared protein concentration at 1.0 mg/mL. Cell lysates were added to Anti-N-Myc Ab 5.0 μg/mL or control mouse IgG 5.0 μg/mL and rotated for 1 h in 4° C. After being rotated, 20 μL Protein G Agarose (Santa Cruz Biotechnology, Inc., Texas, USA) was added to cell lysates and the mixture was rotated overnight at 4° C. Cell lysates were washed by RIPA buffer 3 times and subjected to western blot analysis using anti-N-Myc Ab, and anti-Ubiquitin Ab.

### *In vitro* invasion assay

A transwell chamber was used for the invasion assay. Before preparation, IMR-32 cells (1.0 × 10^5^ cells/well) were treated with or without FNIII14 (50 μg/mL) on FN-coated 6-well plate for 6 days. Culture medium of 10% FBS (200 μL) was added to the lower chamber and the membrane filter (8.0 μm) mentioned above was placed on top. For a basement membrane matrix, EHS-gel Basement membrane Matrix (055-09031; Wako Pure Chemicals Corp., Osaka, Japan) including 2.0 μg/mL FN was adjusted to 600 μg/mL with PBS solution, and 100 μL was applied to the membrane filter. The EHS-gel Basement membrane Matrix was coated by incubation at 37° C in a 5% CO_2_ incubator for 1 h. After removing the supernatant of the gel, IMR-32 cells were pre-treated by vehicle or FNIII14 (5.0 × 10^4^ cells/well), seeded, and incubated at 37°C in a 5% CO_2_ incubator for 24 h. After removing non-permeating cells from the membrane filter with a cotton swab, the invading cells were fixed by a PBS (−) solution including 4% formalin and 10% glycerol with the undersurface as the top at room temperature for 1 h. The invading cells were counted in 4 fields under a 100 × microscope after dyeing the cells with crystal violet stain.

### Animal study

All animal procedures were approved by the Institutional Animal Care and Use Committee (IACUC) of Tokyo University of Science. IMR-32 cells (2.0 × 10^6^ cells/head) suspended in MEM plus 5% FBS with 2.0 mg/mL EHS-gel (200 μL) were subcutaneously injected into the left flanks of 5-week old male Balb/c nude mice (Sankyo Laboratory Service, Tokyo, Japan). Mice bearing established tumors were divided into two groups, ‘Control’ and ‘FNIII14’. The Control group was intravenously administered with saline and intraperitoneally administered with soybean oil, every other day. The FNIII14 group was intravenously administered with saline solution of peptide FNIII14 (100 μg), and intraperitoneally administered with a soybean oil solution of the peptide (500 μg), every other day. These procedures were initially performed for 1 week. Tumor volume (mm^3^) determined by calipers was calculated according to hemi-ellipsoid model: Volume = 2/3 × π × (major axis/2) × (minor axis/2)^2^. After monitoring tumor sizes for 4 weeks, they were removed and homogenized in lysis buffer by BioMasher (Nippi Inc., Tokyo, Japan). Cell lysates were subjected to western blot to detect N-Myc and Aurora A.

### Semi-quantitative PCR

Cell RNA was extracted using the GenElute™ Mammalian Total RNA Miniprep Kit (Sigma-Aldrich Japan) according to the manufacturer’s instructions. After evaluating the RNA concentration by Nano Drop (Thermo Scientific, Danvers, MA), cDNA was gathered by reverse transcription reaction using Quanti Tect^®^ Reverse Transcription (QIAGEN K.K. - Japan, Tokyo, Japan) and amplified in PCR Thermal Cycler Dicer (Takara Bio, Shiga, Japan) using the specific primers (ITGA4 forward: 5′-GCTTCTCAGATCTGCTCGTG-3′, ITGA4 reverse: 5′- GTCACTTCCAACGAGGTTTG-3′, ITGA5 forward: 5′-TGCAGTGTGAGGCTGTGTACA-3′, ITGA5 reverse: 5′-GTGGCCACCTGACGCTCT-3′, ITGAV forward: 5′-GGATTGTTGCTACTGGCTGTTTTGG-3′, ITGAV reverse: 5′-TCCCTTTCTTGTTCTTCTTGAGGTGG-3′, ITGB1 forward: 5′-GAAGGGTTGCCCTCCAGA-3′, ITGB1 reverse: 5′-GCTTGAGCTTCTCTGCTGTT-3′, *GAPDH* forward: 5′-TTCACCACCATGGAGAAGGC-3′, and *GAPDH* reverse: 5′-GGCATGGACTGTGGTCATGA-3′,). The following PCR program was performed: *ITGA4, ITGB1 and GAPDH*, at 94° C for 2 min (initial denaturation), at 94° C for 30 s, at 55° C for 30 s, at 72° C for 40 s, 30 cycles and final extension at 72° C for 7 min.; *ITGA5 and ITGAV*, at 94°C for 2 min (initial denaturation), at 94° C for 30 s, at 60° C for 30 s., at 72° C for 40 s, 30 cycles and final extension at 72° C for 7 min. The PCR products were electrophoresed using 2% agarose-TBE gel including ethidium bromide and developed with a trans-illuminator. Semi-quantitative RT-PCR followed by densitometry scanning. GAPDH served as an internal control.

### Real-time quantitative PCR

RNA extraction and cDNA synthesis were mentioned as above. Real-time polymerase chain reactions were performed on a CFX Connect™ Real-Time PCR system (Bio-Rad Laboratories, Hercules, CA) and using THUNDERBIRD^®^ SYBR^®^ qPCR Mix (Toyobo Co., Ltd., Osaka, Japan) and these specific primers: *MYCN* forward: 5′-CGACCACAAGGCCCTCAGTA-3′, *MYCN* reverse: 5′-CAGCCTTGGTGTTGGAGGAG-3′, *GAPDH* forward: 5′-TTCACCACCATGGAGAAGGC-3′, *GAPDH* reverse: 5′-GGCATGGACTGTGGTCATGA-3′.

### Cell adhesion assay

IMR-32 cells were harvested and suspended (1 × 10^4^ cells/well) in serum-free medium with FNIII14 (50 μg/mL) on fibronectin substrate (2 μg/mL). Cells were incubated in a 96-well plate in a 5% CO_2_ incubator at 37°C for 45 min. Adhered cells were fixed with 4% formalin and 5% glycerol. Fixed cells were counted and the number of spread and attached cells in 4 fields of each well were counted.

### Flow cytometric analysis

Active β1-integrins on the cells were evaluated by flow cytometric analysis using anti-β1-integrin antibody (Clone: AG89) conjugated with phycoerythrin (Medical & Biological Laboratories Co., Ltd., Nagoya, Japan), which recognizes the active conformation-specific epitope of β1-integrin, and BD FACS Aria (BD Bioscience) as previously described.

### Statistical analysis

Data are expressed as means ± standard deviation. Two-tailed Student’s *t*-test or one-way analysis of variance was used to determine statistical differences. Values of *P* < 0.05 were considered statistically significant.

## SUPPLEMENTARY MATERIALS



## Abbreviations

ECM: extracellular matrix
Ab: antibody
mAb: monoclonal antibody
FN: fibronectin
