# Supplementary material for: Inactivation of beta1 integrin induces proteasomal degradation of Myc oncoproteins
Source: Oncotarget. 2019 Aug 13;10(48):4960–72. doi: 10.18632/oncotarget.27131 (PMC6697639; doi:10.18632/oncotarget.27131)
Supplement: Supplementary file 1 [file oncotarget-10-4960-s001.pdf]

Inactivation of beta1 integrin induces proteasomal degradation of Myc oncoproteins

SUPPLEMENTARY MATERIALS

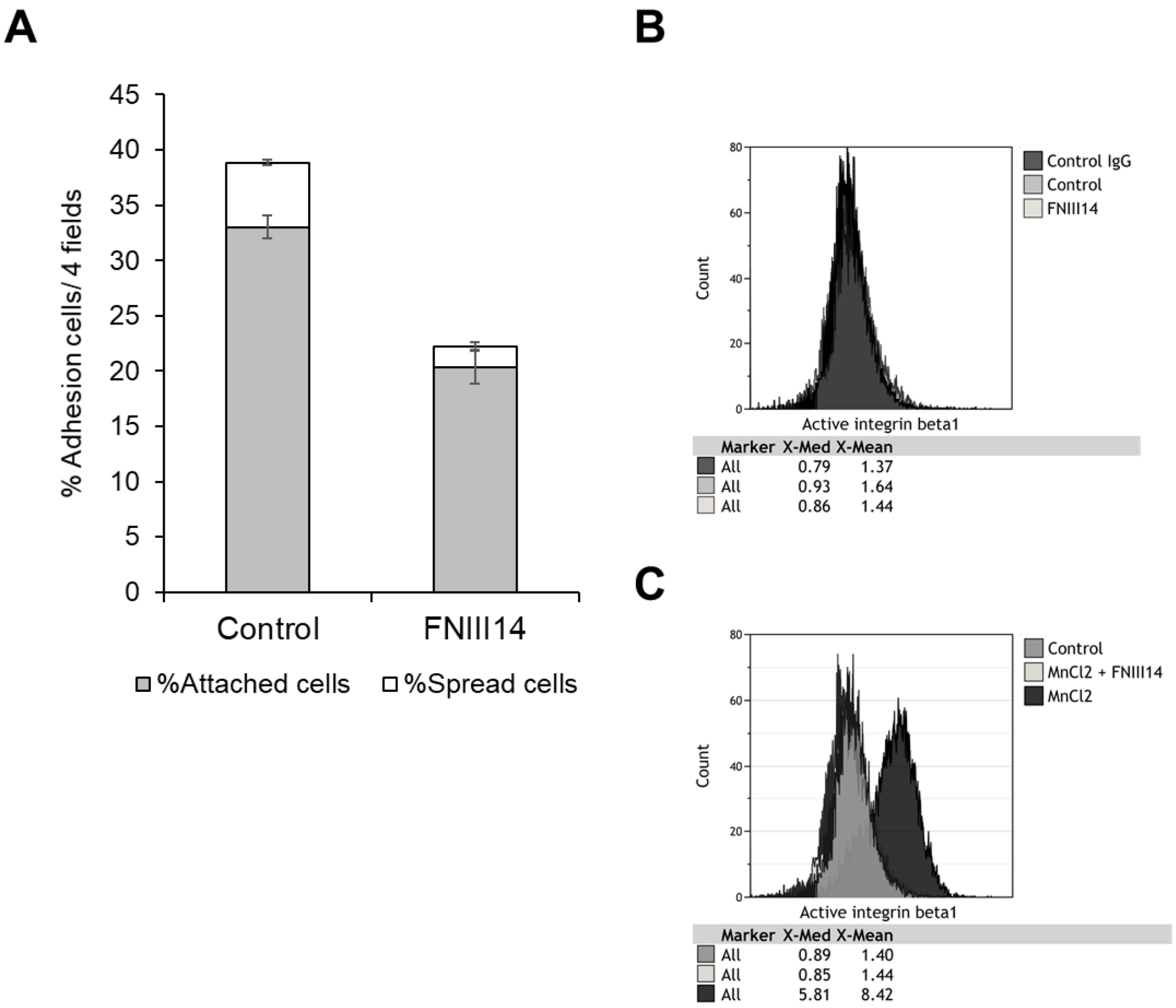

Supplementary Figure 1: FNIII14 inactivates β1- integrins in IMR-32 cells. (**A**) Effect of FNIII14 on cell-adhesion to FN. (**B** and **C**) Effect of FNIII14 on activating state of β1-integrin detected by Flow cytometeric analysis. IMR-32 cells were treated with or without FNIII14 (50 μg/mL) for 45 min (B). IMR-32 cells were treated with $MnCl_2$ (1 mM) in the presence or absence of FNIII14 (50 μg/mL) (C).

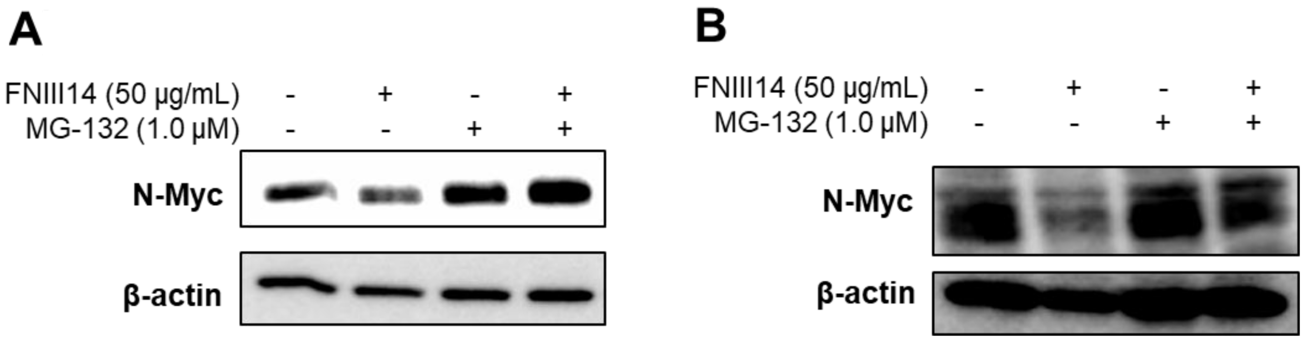

Supplementary Figure 2: Effect of FNIII14 on the expression of N-Myc protein in NB-1 cells (**A**) and KELLY cells (**B**). Cells adhered on fibronectin substrate were stimulated with FNIII14 (50 µg/mL) for 6 days in the presence and absence of MG-132 (1 µM). Cell lysates were subjected to immunoblotting.
